# Network Meta-Analysis of the Antihypertensive Effect of Traditional Chinese Exercises on Patients with Essential Hypertension

**DOI:** 10.1155/2022/9419037

**Published:** 2022-08-17

**Authors:** Lingling Li, Mengdie Yang, Jiao Song, Ying Yu, Hailiang Huang

**Affiliations:** ^1^College of Rehabilitation Medicine, Shandong University of Traditional Chinese Medicine, Jinan, Shandong 250355, China; ^2^Students' Affairs Department, Hebei University of Chinese Medicine, Shijiazhuang, Hebei 050200, China; ^3^College of Health, Shandong University of Traditional Chinese Medicine, Jinan, Shandong 250355, China; ^4^Innovative Institute of Chinese Medicine and Pharmacy, Shandong University of Traditional Chinese Medicine, Jinan, Shandong 250355, China

## Abstract

**Background:**

In recent years, traditional Chinese exercises (TCEs) have been gradually used to reduce the blood pressure levels of patients with essential hypertension. However, there are several types of TCEs, and there is no comparative study on the antihypertensive effects of various TCEs in patients with essential hypertension.

**Objective:**

The objective is to compare the therapeutic effects of Taijiquan (TJQ), Baduanjin (BDJ), Wuqinxi (WQX), and Yijinjing (YJJ) on essential hypertension and provide a reference for clinical treatment and scheme optimization.

**Methods:**

The China National Knowledge Infrastructure (CNKI), Wanfang, China Scientific Journal Database, China Biology Medicine database (CBM), PubMed, Embase, Cochrane Library, and Web of Science databases were searched to collect all randomized controlled trials (RCTs) of TCEs in the treatment of essential hypertension. The search time was from the establishment of each database to November 2021. After data extraction and quality evaluation, the network meta-analysis was performed with Stata 16.0 and ADDIS 1.16.8.

**Results:**

Finally, 45 RCTs involving 3864 patients were included. Network meta-analysis showed that YJJ had the best effect in reducing systolic blood pressure, and the difference was statistically significant [MD = −14.27, 95% CI = (−20.53∼−8.08), *P* < 0.05]. The best probability ranking was YJJ (*P*=0.736) > TJQ (*P*=0.203) > WQX (*P*=0.059) > BDJ (*P*=0.002). In terms of reducing diastolic blood pressure, the treatment effect of YJJ was the best, and the difference was statistically significant [MD = −7.77, 95% CI (−12.19∼−3.33), *P* < 0.05]. The best probability ranking was YJJ (*P*=0.702) > TJQ (*P*=0.178) > WQX (*P*=0.095) > BDJ (*P*=0.025).

**Conclusion:**

The results showed that TCEs significantly reduced systolic and diastolic blood pressure compared with the control group, and YJJ might be the best choice. However, a larger sample, multicenter, double-blinded, high-quality RCTs are needed to make clear conclusions.

## 1. Introduction

Hypertension is a common cardiovascular disease characterized by a continuous increase in systemic arterial blood pressure. Approximately 95% of hypertension cases are called essential hypertension, and the reason is not clear. If blood pressure continues to rise and cannot be controlled in time, serious adverse consequences may occur, such as heart failure, renal failure, and stroke [1]. Epidemiological studies have shown that the number of patients with hypertension in China has reached 245 million [2], and the prevalence rate is 29.6% (95% CI 28.9%∼30.4%), which is increasing year by year [3]. Similarly, it is estimated that by 2025, the number of patients with hypertension in the world will increase by 60%, reaching approximately 15.6 billion [4]. However, only 46% of patients with hypertension are aware of this disease, and only 14% of patients can be effectively controlled [5]. In addition, compared with high-income countries, low-income and middle-income countries are facing higher economic burdens and lower disease control rates [6].

Although most guidelines recommend the use of angiotensin-converting enzyme inhibitors, diuretics, and calcium channel blockers as first-line antihypertensive drugs, long-term use will lead to poor compliance of patients, dizziness, fatigue, orthostatic hypotension, bradycardia, and hypokalemia to different degrees [7]. Traditional Chinese exercises (TCEs) mainly include Baduanjin (BDJ), Taijiquan (TJQ), Wuqinxi (WQX), and Yijinjing (YJJ), which have a good effect on keeping fit and preventing diseases. After inheritance and reform, TCEs are based on the concept of the whole life of the human body, which integrates the concept of health care in traditional Chinese medicine [8]. The “National Clinical Practice Guidelines on the Management of Hypertension in Primary Health Care in China (2020)” issued in December 2020 clearly points out that TCEs such as TJQ and BDJ have antihypertensive effects and can be used as a choice of exercise mode for the management of hypertension at the grassroots level [9].

In recent years, BDJ, TJQ, and other TCEs have been gradually applied to reduce blood pressure levels in patients with essential hypertension. TJQ is gentle, consistent, and flexible and can balance yin and yang, dredge meridians, stabilize emotions, maintain the stability of vascular motor nerves, improve vascular compliance, lower blood pressure, and thus improve the quality of life of patients [10]. BDJ combines breathing toner with psychological adjustment, which can strengthen physical fitness to alleviate brain fatigue, adjust emotions, and promote hypertension patients to produce obvious antihypertensive effects [11]. Based on theories and practical guidance of traditional Chinese medicine, WQX incorporates the traditional movement of tigers, deer, bears, apes, and birds, effectively unblocking the meridians, harmonizing the internal organs, preventing diseases, and extending life expectancy [12]. YJJ mainly promotes the circulation of qi and blood and improves the physiological function of various organs by regulating the heart, qi, and posture [10]. However, there are many kinds of TCEs, and there is still a lack of comparative studies on the antihypertensive effects of different TCEs on essential hypertension patients. The relative effectiveness of TCEs cannot be evaluated, which is not conducive to the promotion and application of TCEs in lowering blood pressure and the selection of diagnosis and treatment schemes.

Network meta-analysis was developed from a traditional meta-analysis. The greatest advantage of network meta-analysis was that different interventions for the treatment of similar diseases can be summarized for quantitative analysis, sorted according to a certain result index, and then the optimal treatment plan can be selected [13]. Therefore, the purpose of this study was to compare the curative effects of four TCEs on essential hypertension by using the network meta-analysis method according to the probability ranking to provide reliable evidence-based medicine for clinical treatment and optimal scheme selection.

## 2. Materials and Methods

### 2.1. Search Strategy

The China National Knowledge Infrastructure (CNKI), Wanfang, China Scientific Journal Database, China Biology Medicine database (CBM), PubMed, Embase, Cochrane Library, and Web of Science databases were searched to collect all randomized controlled trials (RCTs) of traditional Chinese exercises in the treatment of essential hypertension. The search time was from the establishment of each database to November 2021.

Taking the Embase database as an example, the specific retrieval formula is (“traditional Chinese exercises”: ti, ab, kw OR “traditional fitness exercise”: ti, ab, kw OR “traditional exercise therapy”: ti, ab, kw OR “traditional exercise”: ti, ab, kw OR “health qigong”: ti, ab, kw OR “qigong”: ti, ab, kw OR “tai chi exercise”: ti, ab, kw OR “tai chi”: ti, ab, kw OR “taijiquan”: ti, ab, kw OR “baduanjin”: ti, ab, kw OR “eight section brocade”: ti, ab, kw OR “wuqinxi”: ti, ab, kw OR “five-animal exercises”: ti, ab, kw OR “yijinjing”: ti, ab, kw) AND (“essential hypertension”: ti, ab, kw OR “hypertension”: ti, ab, kw).

### 2.2. Inclusion Criteria

#### 2.2.1. Types of Study

Types of studies are RCTs of TCEs for essential hypertension.

#### 2.2.2. Types of Participants

The patients should be definitively diagnosed with essential hypertension, and the age, sex, and source of cases are not limited. They should meet the global hypertension practice guide or other relevant authoritative diagnostic criteria formulated by the International Society of Hypertension in 2020 [14].

#### 2.2.3. Types of Intervention

Patients in the treatment group received any kind of TCEs (TJQ, BDJ, WAX, and YJJ), while patients in the control group did not receive any exercise intervention or directly compared the above four TCEs.

#### 2.2.4. Types of Outcomes

Types of outcomes are Systolic blood pressure (SBP) and diastolic blood pressure (DBP).

### 2.3. Exclusion Criteria

Baselines are incomparable or not reported in the studies; there was no clear diagnosis of essential hypertension or secondary hypertension in the studies; the included cases were severe hypertension, hypertension crisis, severe heart failure, liver and kidney dysfunction, and other major diseases; non-RCTs such as self-control, cohort study, and case-control study; studies on the combination of intervention and other therapeutic methods; studies without corresponding outcomes; the information was incomplete, and the original data and full text could not be obtained after contacting the author; studies with inaccurate designs or improper statistical methods; and meeting abstracts, protocols, animal experiments, and reviews.

### 2.4. Literature Selection and Data Extraction

Duplicate documents were eliminated by EndNote X 9 software, and then the titles and abstracts were read independently by two reviewers for preliminary screening. Then, the full text of studies that may meet the inclusion criteria was rescreened. The following data were extracted from the included studies: first author, publication year, sample size, intervention measures, intervention period, outcomes, quality evaluation, and adverse reactions.

### 2.5. Risk of Bias and Quality Assessment

According to the risk bias assessment tool of the systematic review provided by the Cochrane collaboration network, two reviewers evaluated the quality of the included studies. If there were any differences in the assessment results, they were decided by the third reviewer. The assessment items included random sequence generation, concealment of distribution, blinding method, data integrity, selective reporting, and other biases. The quality of the included studies was assessed according to three options: high risk, low risk, and unclear. If the above assessment items were low risk, the evidence grade was A; if some assessment items were low risk, the evidence grade was B; and if all assessment items were high risk, the evidence grade was C. To ensure the quality of the included studies, the studies with an evidence grade of C were excluded from this study.

### 2.6. Statistical Analysis

After data extraction and quality assessment of the included studies, Stata 16.0 and ADDIS 1.16.8 software were used to conduct a network meta-analysis based on the Markov Chain Monte Carlo algorithm. SBP and DBP are continuous variables, so the effect value and effect quantity are expressed by the mean difference (MD) and 95% confidence interval (CI), respectively. When the 95% CI did not contain 0, there was a significant difference between the experimental group and the control group (*P* < 0.05). The node-split model was used to test the inconsistency. If there was no significant difference (*P* > 0.05), it indicates that the heterogeneity of the included studies was small, so the consistency model was used for network meta-analysis. In contrast, the inconsistency model was used for network meta-analysis. The potential scale reduction factor (PSRF) is calculated by comparing the intrachain and interchain differences. PSRF is close to 1, which indicates that the convergence is good and the consistency model analysis results are reliable.

## 3. Results

### 3.1. Literature Selection

**Figure 1:**
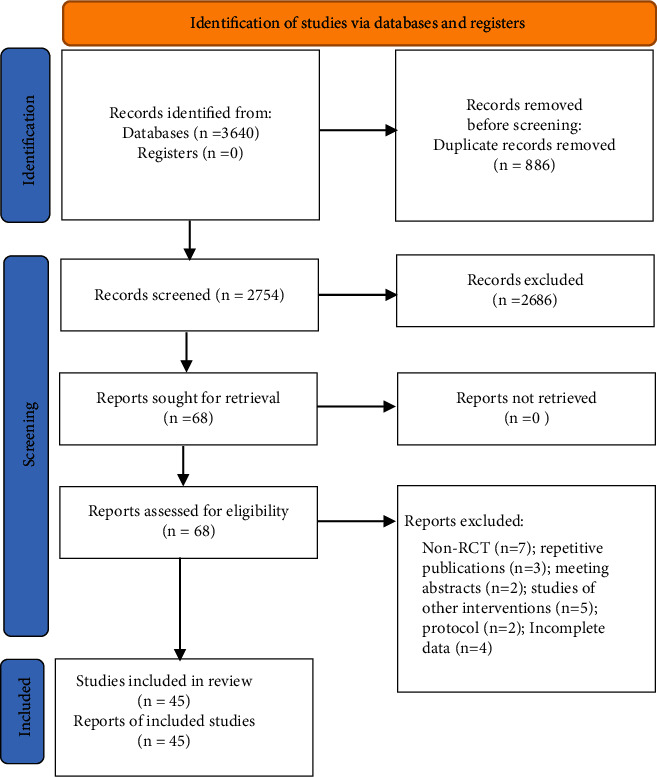
The literature screening process.

A total of 3640 articles were retrieved from the databases, 2754 articles were obtained after preliminary screening, and RCTs [15–30], [31–45], [46–59] were ultimately included after rescreening. The literature screening process is shown in Figure 1.

### 3.2. Basic Information of the Included Studies

In the end, a total of 45 RCTs [15–59] were included, 44 of which were double-arm trials [15–27, 29–59] and 1 RCT [28] was a three-arm trial. There were 3864 patients in total, 1962 in the experimental group and 1902 in the control group. The intervention measures of 23 RCTs [15–27, 29–38] were TJQ, 5 RCTs [39–43] were WQX, 4 RCTs [44–47] were YJJ, and 12 RCTs [48–59] were BDJ. In addition, the intervention measures of the three-arm RCT were TJQ and BDJ.

### 3.3. Bias Risk Assessment

The evidence levels of 45 RCTs of included studies were all B, and the baseline was reported, referring to random grouping. Thirteen RCTs [17, 19, 25, 26, 29, 31, 37, 38, 48, 50, 52, 55, 56] described the specific method of random sequence generation, and only 3 RCTs [31, 37, 38] were hidden groups. The study itself did not blind researchers and patients, and 19 RCTs [15, 17–19, 23, 24, 30, 31, 33, 36–38, 43–45, 47, 49, 53, 59] were blinded result assessments. As shown in Table 1, the data of the included studies are complete, and there are no selective reporting or other bias risks.

### 3.4. Network Meta-Analysis

**Figure 2:**
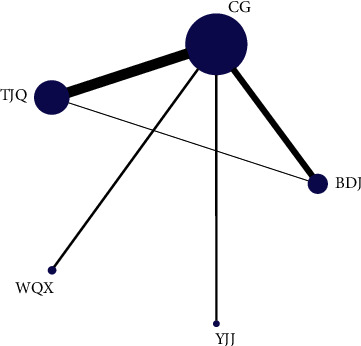
The network relationship of TCEs for essential hypertension. PS: TJQ, Taijiquan; BDJ, Baduanjin; WQX, Wuqinxi; YJJ, Yijinjing; CG, control group.

**Figure 3:**
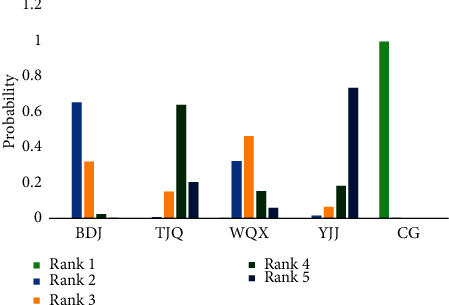
The probability ranking of TCEs for essential hypertension on SBP score. PS: TJQ, Taijiquan; BDJ, Baduanjin; WQX, Wuqinxi; YJJ, Yijinjing; CG, control group.

**Figure 4:**
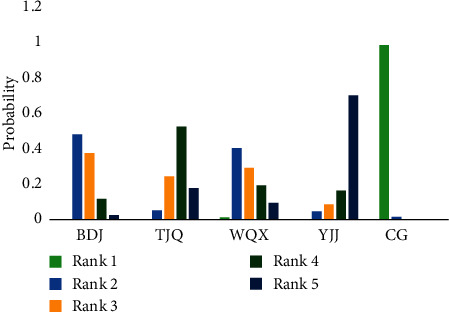
The probability ranking of TCEs for essential hypertension on DBP score. PS: TJQ, Taijiquan; BDJ, Baduanjin; WQX, Wuqinxi; YJJ, Yijinjing; CG, control group.

The network relationship of TCEs for essential hypertension is shown in Figure 2. The connection between the two blue balls indicates that RCTs can be directly compared between the two interventions, while the lack of connection indicated that RCTs cannot be directly compared, and the control group could be used as a reference for indirect comparison. The thickness of the connection between the two blue balls represents the number of RCTs compared between the two interventions. The size of the blue ball represents the number of participants in each intervention. In this study, there was a closed loop of direct evidence and indirect evidence, and the node-split model was used to test the inconsistency. The results showed that *P* = 0.6025 > 0.05, which indicates that the heterogeneity of the included studies was small, so the consistency model was adopted. The results of the network meta-analysis are shown in Table 2, and the probability rankings are shown in Figures 3 and 4 and Table 3. SBP and DBP negative scoring outcome indicators were ranked with the probability by Rank N. A higher Rank N represents great outcome indicators. The PSRF was close to 1.00, which indicated good convergence.

#### 3.4.1. SBP

The SBP score was reported in 45 RCTs [15–30], [31–45], [46–59] involving 4 TCEs. There were significant differences between the four TCE groups and the control group in reducing diastolic blood pressure scores BDJ [MD = −7.26, 95% CI (−10.59∼−3.90), *P* < 0.05], TJQ [MD = −11.81, 95% CI (−14.48∼−9.09), *P* < 0.05], WQX [MD = −8.72, 95% CI (−14.6∼−2.79), *P* < 0.05], and YJJ [MD = −14.27, 95% CI = (−20.53∼−8.08), *P* < 0.05]^12^. As shown in Figure 3, YJJ had 0.736 probability in ranking 5 among the treatments, which meant YJJ was more likely to yield the best treatment outcome on the SBP score. The probability ranking was YJJ (*P*=0.736) > TJQ (*P*=0.203) > WQX (*P*=0.059) > BDJ (*P*=0.002).

#### 3.4.2. DBP

The DBP score was reported in 45 RCTs [15–30],[31–45],[46–59] involving 4 TCEs. There were significant differences between the four TCE groups and the control group in reducing diastolic blood pressure scores BDJ [MD = −4.35, 95% CI (−6.90∼−1.78), *P* < 0.05], TJQ [MD = −6.04, 95% CI (−7.97∼−4.11), *P* < 0.05], WQX [MD = −4.64, 95% CI (−8.75∼−0.55), *P* < 0.05], and YJJ [MD = −7.77, 95% CI (−12.19∼−3.33), *P* < 0.05]. [12] As shown in Figure 4, YJJ had 0.702 probability in ranking 5 among the treatments, which meant YJJ was more likely to yield the best treatment outcome on the DBP score. The probability ranking was YJJ (*P*=0.702) > TJQ (*P*=0.178) > WQX (*P*=0.095) > BDJ (*P*=0.025).

## 4. Discussion

Hypertension is a common and frequently occurring disease, an important cause and risk factor for various cardiovascular and cerebrovascular diseases, and one of the major causes of death of cardiovascular and cerebrovascular diseases. Essential hypertension is characterized by slow onset and lack of characteristic symptoms, which are mainly higher than normal blood pressure. Its goal is to minimize the total risk of cardiovascular disease morbidity and mortality and improve physical activity and quality of life. [60] In other words, it is of great significance to find other methods to control blood pressure in addition to drugs to reduce the mortality of cardiovascular diseases and improve the quality of life of residents. At present, the TCEs promoted by the General Administration of Sport of China mainly include BDJ, TJQ, YJJ, and WQX, which are easy to learn, are easy for patients to accept, and have moderate exercise intensity. TCEs have been inherited and reformed based on the holistic view of human life, combining Chinese medicine health care with Chinese medicine health preservation, which plays a very good role in strengthening the body and preventing disease [8, 12]. In recent years, it has achieved remarkable curative effects in treating essential hypertension and gradually gained popularity [61].

The purpose of this study was to objectively compare the effects of 4 TCEs on essential hypertension by network meta-analysis and probability ranking of 45 RCTs with SBP and DBP scores as outcomes. The results showed that TCEs significantly reduced SBP and DBP scores compared with the control group, and YJJ might be the best choice. This study provides an evidence-based medicine reference for patients with essential hypertension to choose different TCEs, and it has a certain reference value for guiding patients with essential hypertension to choose the best scheme in the future.

TJQ, BDJ, YJJ, and other TCEs combine music with exercise and guide action with consciousness so that form and spirit can be interlinked and thus spirit can reach the realm. Through manipulation exercise, the function of viscera can be stimulated, and the qi, blood, and body fluid can be reconciled. The internal yin and yang rise and fall orderly, and the external muscles and bones are luxuriant so that the human body tends to relax and achieve the purpose of lowering blood pressure [62]. “Normalization, stability, and comprehensiveness” are the three principles of treatment for patients with hypertension [7, 9]. Exercise therapy mainly focuses on lowering peripheral blood pressure and advocates aerobic exercise with low and medium intensity. TCEs can not only reduce blood pressure to a certain extent but also regulate the body's reaction to exercise, thus promoting the recovery of patients. [60] Patients with hypertension can choose the corresponding TCEs according to their own actual situation, persistently eliminate the disease and improve their quality of life. In addition, it is also very important to prevent and treat hypertension and effectively reduce the risk of elderly individuals and chronic diseases. It is an urgent problem to popularize the concept of “prevention is more important than cure” and even to implant it into the national consciousness.

YJJ pays more attention to the exercise of posture, breathing, and mind than other TCEs and exercises according to the movement of the twelve meridians and Ren and Du—two Mai. That is, every potential method corresponds to dredging a meridian. [63] Hong's research [44] shows that through a specific posture, the whole muscle and vein are in a highly active state, and the qi in the body is urged to move according to a specific trajectory to achieve the effect of dredging meridians, activating qi and blood, preventing diseases, and keeping fit. [47] Studies have also shown that exercising the vagus nerve for 1 hour in the morning can improve myocardial ischemia and hypoxia and reduce the occurrence of diseases such as hypertension and atherosclerosis, which may be related to maintaining a high level of vagus nerve tension, thus strengthening the regulation of autonomic nerve function. [64] At present, there are relatively few studies on YJJ in treating essential hypertension, and its mechanism of action is not clear, which may become a breakthrough in future research.

Although this study follows the Preferred Reporting Items for Systematic Reviews and Network Meta-Analysis [65, 66], there are still some limitations. All the included studies were published in English and Chinese, and a lack of relevant gray literature may lead to selection bias of the literature. The sample size of the included studies was small, and the quality evaluation evidence level was medium. Only 3 RCTs [31, 37, 38] used opaque envelopes to hide the study groups. This study is difficult for blind researchers and patients, so it does not rule out the possibility of bias. The stage of hypertension, intervention period (6 [26]–48 weeks [33, 36, 51]), intervention frequency, duration of each intervention, and subjectivity of outcome index measurement may affect the results of network meta-analysis.

## 5. Conclusion

In conclusion, the results showed that TCEs significantly reduced systolic and diastolic blood pressure compared with the control group, and YJJ might be the best choice. However, a larger sample, multicenter, double-blinded, high-quality RCTs are needed to make clear conclusions.

## Figures and Tables

**Table 1:** Bias risk assessment.

Studies	Random sequence generation	Concealment of distribution	Blind method of researchers and patients	Blind method of assessments	Data integrity	Selective reporting	Other biases
Luo [15]	Unclear	Unclear	High risk	Low risk	Low risk	Low risk	Low risk
Zhou [16]	Unclear	Unclear	High risk	Unclear	Low risk	Low risk	Low risk
Mao and Shao [17]	Low risk	Unclear	High risk	Low risk	Low risk	Low risk	Low risk
Sun [18]	Unclear	Unclear	High risk	Low risk	Low risk	Low risk	Low risk
Jing [19]	Low risk	Unclear	High risk	Low risk	Low risk	Low risk	Low risk
Wang et al. [20]	Unclear	Unclear	High risk	Unclear	Low risk	Low risk	Low risk
Chen and Lv [21]	Unclear	Unclear	High risk	Unclear	Low risk	Low risk	Low risk
Sun and Sun [22]	Unclear	Unclear	High risk	Unclear	Low risk	Low risk	Low risk
Xie and Bai [23]	Unclear	Unclear	High risk	Low risk	Low risk	Low risk	Low risk
Zheng et al. [24]	Unclear	Unclear	High risk	Low risk	Low risk	Low risk	Low risk
Xu [25]	Low risk	Unclear	High risk	Unclear	Low risk	Low risk	Low risk
Jin and Pang [26]	Low risk	Unclear	High risk	Unclear	Low risk	Low risk	Low risk
Liu [27]	Unclear	Unclear	High risk	Unclear	Low risk	Low risk	Low risk
Shi and Miao [28]	Unclear	Unclear	High risk	Unclear	Low risk	Low risk	Low risk
Liu et al. [29]	Low risk	Unclear	High risk	Unclear	Low risk	Low risk	Low risk
Shou et al. [30]	Unclear	Unclear	High risk	Low risk	Low risk	Low risk	Low risk
Zhong [31]	Low risk	Low risk	High risk	Low risk	Low risk	Low risk	Low risk
Tsai et al. [32]	Unclear	Unclear	High risk	Unclear	Low risk	Low risk	Low risk
Wolf et al. [33]	Unclear	Unclear	High risk	Low risk	Low risk	Low risk	Low risk
Lo et al. [34]	Unclear	Unclear	High risk	Unclear	Low risk	Low risk	Low risk
Nguyen and Kruse [35]	Unclear	Unclear	High risk	Unclear	Low risk	Low risk	Low risk
Sun and Buys [36]	Unclear	Unclear	High risk	Low risk	Low risk	Low risk	Low risk
Chan et al. [37]	Low risk	Low risk	High risk	Low risk	Low risk	Low risk	Low risk
Ma et al. [38]	Low risk	Low risk	High risk	Low risk	Low risk	Low risk	Low risk
Hong [39]	Unclear	Unclear	High risk	Unclear	Low risk	Low risk	Low risk
Lin and Huang [40]	Unclear	Unclear	High risk	Unclear	Low risk	Low risk	Low risk
Li et al. [41]	Unclear	Unclear	High risk	Unclear	Low risk	Low risk	Low risk
Shen et al. [42]	Unclear	Unclear	High risk	Unclear	Low risk	Low risk	Low risk
Xin [43]	Unclear	Unclear	High risk	Low risk	Low risk	Low risk	Low risk
Hong and Wang [44]	Unclear	Unclear	High risk	Low risk	Low risk	Low risk	Low risk
Yao [45]	Unclear	Unclear	High risk	Low risk	Low risk	Low risk	Low risk
Feng et al. [46]	Unclear	Unclear	High risk	Unclear	Low risk	Low risk	Low risk
Lu [47]	Unclear	Unclear	High risk	Low risk	Low risk	Low risk	Low risk
Pan and Feng [48]	Low risk	Unclear	High risk	Unclear	Low risk	Low risk	Low risk
Chen and Zhou [49]	Unclear	Unclear	High risk	Low risk	Low risk	Low risk	Low risk
Chen [50]	Low risk	Unclear	High risk	Unclear	Low risk	Low risk	Low risk
Yu [51]	Unclear	Unclear	High risk	Unclear	Low risk	Low risk	Low risk
Lin and He [52]	Low risk	Unclear	High risk	Unclear	Low risk	Low risk	Low risk
Yang [53]	Unclear	Unclear	High risk	Low risk	Low risk	Low risk	Low risk
Liu [54]	Unclear	Unclear	High risk	Unclear	Low risk	Low risk	Low risk
He [55]	Low risk	Unclear	High risk	Unclear	Low risk	Low risk	Low risk
Dong and Zhang [56]	Low risk	Unclear	High risk	Unclear	Low risk	Low risk	Low risk
Liang et al. [57]	Unclear	Unclear	High risk	Unclear	Low risk	Low risk	Low risk
Lin and Yan [58]	Unclear	Unclear	High risk	Unclear	Low risk	Low risk	Low risk
Dong et al. [59]	Unclear	Unclear	High risk	Low risk	Low risk	Low risk	Low risk

**Table 2:** Network meta-analysis results of TCEs for essential hypertension.

BDJ	1.69 (−1.47, 4.85)	0.30 (−4.51, 5.11)	3.42 (−1.70, 8.54)	−4.35 (−6.90, −1.78)
4.56 (0.32, 8.75)	TJQ	−1.40 (−5.93, 3.15)	1.73 (−3.12, 6.54)	−6.04 (−7.97, −4.11)
1.47 (−5.36, 8.23)	−3.10 (−9.58, 3.40)	WQX	3.12 (−2.96, 9.15)	−4.64 (−8.75, −0.55)
7.02 (−0.04, 14.12)	2.45 (−4.29, 9.31)	5.55 (−2.99, 14.16)	YJJ	−7.77 (−12.19, −3.33)
−7.26 (−10.59, −3.90)	−11.81 (−14.48, −9.09)	−8.72 (−14.6, −2.79)	−14.27 (−20.53, −8.08)	Control group

^*∗*^Blank part is SBP and the gray part is DBP. PS: TJQ, Taijiquan; BDJ, Baduanjin; WQX, Wuqinxi; YJJ, Yijinjing; CG, control group.

**Table 3:** The best probability ranking of TCEs for essential hypertension.

Interventions	SBP	DBP
BDJ	0.002	0.025
TJQ	0.203	0.178
WQX	0.059	0.095
YJJ	0.736	0.702

PS: TJQ, Taijiquan; BDJ, Baduanjin; WQX, Wuqinxi; YJJ, Yijinjing; CG, control group.

## Data Availability

The original contributions presented in the study are included in the article/supplementary material, further inquiries can be directed to the corresponding author.

## Supplementary Materials

Supplementary Materials S1: PRISMA_2020_checklist.
