# Supplementary material for: Network Meta-Analysis of the Antihypertensive Effect of Traditional Chinese Exercises on Patients with Essential Hypertension
Source: J Healthc Eng. 2022 Aug 17;2022:9419037. doi: 10.1155/2022/9419037 (PMC9402382; doi:10.1155/2022/9419037)
Supplement: Supplementary Materials — S1: PRISMA_2020_checklist. [file 9419037.f1.docx]

| **Section and Topic** | **Item #** | **Checklist item** | **Location where item is reported** |
| --- | --- | --- | --- |
| **TITLE** | | |  |
| Title | 1 | Network meta-analysis of the antihypertensive effect of traditional Chinese exercises on patients with essential hypertension | Page 1 |
| **ABSTRACT** | | |  |
| Abstract | 2 | Background: In recent years, traditional Chinese exercises (TCEs) have been gradually used to reduce the blood pressure levels of patients with essential hypertension. However, there are several types of TCEs, and there is no comparative study on the antihypertensive effects of various TCEs in patients with essential hypertension.  Objective: To compare the therapeutic effects of Taijiquan (TJQ), Baduanjin (BDJ), Wuqinxi (WQX) and Yijinjing (YJJ) on essential hypertension and provide a reference for clinical treatment and scheme optimization.  Methods: The CNKI, Wanfang, VIP, Chinese biomedical literature, PubMed, Embase, Cochrane Library, and Web of Science databases were searched to collect all randomized controlled trials (RCTs) of TCEs in the treatment of essential hypertension. The search time was from the establishment of each database to November 2021. After data extraction and quality evaluation, the network meta-analysis was performed with Stata 16.0 and ADDIS 1.16.8.  Results: Finally, 45 RCTs involving 3864 patients were included. Network meta-analysis showed that YJJ had the best effect in reducing systolic blood pressure, and the difference was statistically significant [MD=-14.27, 95% CI = (-20.53 ~ -8.08), P<0.05]. The best probability ranking was YJJ (P = 0.736) > TJQ (P = 0.203) > WQX (P = 0.059) > BDJ (P=0.002). In terms of reducing diastolic blood pressure, the treatment effect of YJJ was the best, and the difference was statistically significant [MD=-7.77, 95% CI (-12.19 ~ -3.33), P<0.05]. The best probability ranking was YJJ (P=0.702) > TJQ (P=0.178) > WQX (P=0.095) > BDJ (P=0.025).  Conclusion: The results showed that TCEs significantly reduced systolic and diastolic blood pressure compared with the control group, and YJJ might be the best choice. However, more large sample, multicenter, double-blind, high-quality RCTs are needed to make clear conclusions.  Relevance to clinical practice: TCEs are easy to learn, acceptable to patients and of moderate intensity, and can be used as non-pharmacological intervention methods in hypertensive patients. This study showed that TCEs is an effective and suitable exercise nursing method, which can reduce systolic and diastolic blood pressure of patients with essential hypertension. | 1-2 |
| **INTRODUCTION** | | |  |
| Rationale | 3 | Hypertension is a common cardiovascular disease characterized by a continuous increase in systemic arterial blood pressure. Approximately 95% of hypertension cases are called essential hypertension, and the reason is not clear. If blood pressure continues to rise and cannot be controlled in time, serious adverse consequences may occur, such as heart failure, renal failure, and stroke.1 Epidemiological studies have shown that the number of patients with hypertension in China has reached 245 million,2 and the prevalence rate is 29.6% (95% CI 28.9%~30.4%), which is increasing year by year.3 Similarly, it is estimated that by 2025, the number of patients with hypertension in the world will increase by 60%, reaching approximately 15.6 billion.4 However, only 46% of patients with hypertension are aware of this disease, and only 14% of patients can be effectively controlled.5 In addition, compared with high-income countries, low-income and middle-income countries are facing higher economic burdens and lower disease control rates.6  Although most guidelines recommend the use of angiotensin-converting enzyme inhibitors, diuretics, and calcium channel blockers as first-line antihypertensive drugs, long-term use will lead to poor compliance of patients, dizziness, fatigue, orthostatic hypotension, bradycardia, and hypokalaemia to different degrees.7 Traditional Chinese exercises (TCEs) mainly include Baduanjin (BDJ), Taijiquan (TJQ), Wuqinxi (WQX) and Yijinjing (YJJ), which have a good effect on keeping fit and preventing diseases. After inheritance and reform, TCEs are based on the concept of the whole life of the human body, which integrates the concept of health care in traditional Chinese medicine.8 The “National Clinical Practice Guidelines on the Management of Hypertension in Primary Health Care in China (2020)” issued in December 2020 clearly points out that TCEs such as TJQ and BDJ have antihypertensive effects and can be used as a choice of exercise mode for the management of hypertension at the grassroots level.9  In recent years, BDJ, TJQ and other TCEs have been gradually applied to reduce blood pressure levels in patients with essential hypertension. However, there are many kinds of TCEs, and there is still a lack of comparative studies on the antihypertensive effects of different TCEs on essential hypertension patients. The relative effectiveness of TCEs cannot be evaluated, which is not conducive to the promotion and application of TCEs in lowering blood pressure and the selection of diagnosis and treatment schemes.  Network meta-analysis was developed from traditional meta-analysis. The greatest advantage of network meta-analysis was that different interventions for the treatment of similar diseases can be summarized for quantitative analysis, sorted according to a certain result index, and then the optimal treatment plan can be selected. | 2-3 |
| Objectives | 4 | Therefore, the purpose of this study was to compare the curative effects of four TCEs on essential hypertension by using the network meta-analysis method according to the probability ranking to provide reliable evidence-based medicine evidence for clinical treatment and optimal scheme selection. | 3 |
| **METHODS** | | |  |
| Eligibility criteria | 5 | 2.2.1. Types of study: RCTs of TCEs for Essential Hypertension.  2.2.2. Type of participants: The patients should be definitively diagnosed with essential hypertension, and the age, sex and source of cases are not limited. They should meet the global hypertension practice guide or other relevant authoritative diagnostic criteria formulated by the International Society of Hypertension in 2020.11  2.2.3. Type of intervention: Patients in the treatment group received any kind of TCE (TJQ, BDJ, WAX, YJJ), while patients in the control group did not receive any exercise intervention or directly compared the above four TCEs.  2.2.4. Types of outcomes: systolic blood pressure (SBP) and diastolic blood pressure (DBP). | 4-5 |
| Information sources | 6 | The CNKI, Wanfang, VIP, Chinese biomedical literature, PubMed, Embase, Cochrane Library, and Web of Science databases were searched to collect all randomized controlled trials (RCTs) of traditional Chinese exercises in the treatment of essential hypertension. The search time was from the establishment of each database to November 2021. | 4 |
| Search strategy | 7 | Taking the Embase database as an example, the specific retrieval formula is (“traditional Chinese exercises”: ti, ab, kw OR “traditional fitness exercise”: ti, ab, kw OR “traditional exercise therapy”: ti, ab, kw OR “traditional exercise”: ti, ab, kw OR “health qigong”: ti, ab, kw OR “qigong”: ti, ab, kw OR “tai chi exercise”: ti, ab, kw OR “tai chi”: ti, ab, kw OR “taijiquan”: ti, ab, kw OR “baduanjin”: ti, ab, kw OR “eight section brocade”: ti, ab, kw OR “wuqinxi”: ti, ab, kw OR “five-animal exercises”: ti, ab, kw OR “yijinjing”: ti, ab, kw) AND (“essential hypertension”: ti, ab, kw OR “hypertension”: ti, ab, kw). | 4 |
| Selection process | 8 | Two researchers independently screened the literature and extracted and cross-checked the data. If there was any disagreement, it was discussed with a third researcher. | 5 |
| Data collection process | 9 | Two researchers independently screened the literature and extracted and cross-checked the data. If there was any disagreement, it was discussed with a third researcher. | 5 |
| Data items | 10 | The following data were extracted from the included studies: first author, publication year, sample size, intervention measures, intervention period, outcomes, quality evaluation and adverse reactions. | 5 |
| Study risk of bias assessment | 11 | According to the risk bias assessment tool of systematic review provided by the Cochrane collaboration network, two authors evaluated the quality of the included studies. If there were any differences in the assessment results, they were decided by the third author. The assessment items included random sequence generation, concealment of distribution, blinding method, data integrity, selective reporting and other biases. The quality of the included studies was assessed according to three options: high risk, low risk and unclear. If the above assessment items were low risk, the evidence grade was A; if some assessment items were low risk, the evidence grade was B; and if all assessment items were high risk, the evidence grade was C. To ensure the quality of the included studies, the studies with an evidence grade of C were excluded from this study. | 5-6 |
| Effect measures | 12 | the outcomes of the included studies were continuous variables, so the weighted mean difference (MD) and 95% confidence interval (CI) were used as the effect values. The test level α = 0.05. | 6 |
| Synthesis methods | 13 | After data extraction and quality assessment of the included studies, Stata 16.0 and ADDIS 1.16.8 software were used to conduct network meta-analysis based on the Markov Chain-Monte Carlo algorithm. SBP and DBP are continuous variables, so the effect value and effect quantity are expressed by the mean difference (MD) and 95% confidence interval (CI), respectively. When the 95% CI did not contain 0, there was a significant difference between the experimental group and the control group (P<0.05). The node-split model was used to test the inconsistency. If there was no significant difference (P>0.05), it indicates that the heterogeneity of the included studies was small, so the consistency model was used for network meta-analysis. In contrast, the inconsistency model was used for network meta-analysis. The potential scale reduction factor (PSRF) is calculated by comparing the intrachain and interchain differences. PSRF is close to 1, which indicates that the convergence is good and the consistency model analysis results are reliable. | 6 |
| Reporting bias assessment | 14 | Not suitable |  |
| Certainty assessment | 15 | Not suitable |  |
| **RESULTS** | | |  |
| Study selection | 16 | A total of 3640 articles were retrieved from the databases, 2754 articles were obtained after preliminary screening, and 45 RCTs12-56 were ultimately included after rescreening. The literature screening process was shown in Figure 1. | 6 |
| Study characteristics | 17 | In the end, a total of 45 RCTs12-56 were included, 44 of which were double-arm trials,12-24,26-56 and 1 RCT25 was a three-arm trial. There were 3864 patients in total, 1962 in the experimental group and 1902 in the control group. The intervention measures of 23 RCTs12-24,26-35 were TJQ, 5 RCTs36-40 were WQX, 4 RCTs41-44 were YJJ, and 12 RCTs45-56 were BDJ. In addition, the intervention measures of the three-arm RCT were TJQ and BDJ. | 7 |
| Risk of bias in studies | 18 | The evidence levels of 45 RCTs of included studies were all B, and the baseline was reported, referring to random grouping. Thirteen RCTs14,16,22-23,26,28,34-35,45,47,49,52-53 described the specific method of random sequence generation, and only 3 RCTs28,34-35 were hidden groups. The study itself did not blind researchers and patients, and 19 RCTs12,14-16,20-21,27-28,30,33-35,40-42,44,46,50,56 were blinded result assessments. As shown in Table 1, the data of the included studies are complete, and there are no selective reporting or other bias risks. | 7 |
| Results of individual studies | 19 | Figure 1 |  |
| Results of syntheses | 20 | The network relationship of TCEs for essential hypertension was shown in Figure 2. The connection between the two blue balls indicates that RCTs can be directly compared between the two interventions, while the lack of connection indicated that RCTs cannot be directly compared, and the control group could be used as a reference for indirect comparison. The thickness of the connection between the two blue balls represented the number of RCTs compared between the two interventions. In this study, there was a closed loop of direct evidence and indirect evidence, and the node-split model was used to test the inconsistency. The results showed that *P*=0.6025>0.05, which indicates that the heterogeneity of the included studies was small, so the consistency model was adopted. The results of the network meta-analysis were shown in Table 2, and the probability ranking were shown in Figures 3 and Table 3. The PSRF was close to 1.00, which indicated good convergence.  **3.4.1** **SBP**  The SBP score was reported in 45 RCTs^12-56^ involving 4 TCEs. There were significant differences between the four TCE groups and the control group in reducing diastolic blood pressure scores [MD=-7.26, 95% CI (-10.59 ~ -3.90), *P*<0.05], [MD=-11.81, 95% CI (-14.48 ~ -9.09), *P*<0.05], [MD=-8.72, 95% CI (-14.6 ~ -2.79), *P*<0.05], [MD=-14.27, 95% CI = (-20.53 ~ -8.08), *P*<0.05]. The probability ranking was YJJ (*P*=0.736) > TJQ (*P*=0.203) > WQX (*P*=0.059) > BDJ (*P*=0.002).  **3.4.2 DBP**  The DBP score was reported in 45 RCTs^12-56^ involving 4 TCEs. There were significant differences between the four TCE groups and the control group in reducing diastolic blood pressure scores [MD=-4.35, 95% CI (-6.90 ~ -1.78), *P*<0.05], [MD=-6.04, 95% CI (-7.97 ~ -4.11), *P*<0.05], [MD=-4.64, 95% CI (-8.75 ~ -0.55), *P*<0.05], [MD=-7.77, 95% CI (-12.19 ~ -3.33), *P*<0.05]. The probability ranking was YJJ (*P*=0.702) > TJQ (*P*=0.178) > WQX (*P*=0.095) > BDJ (*P*=0.025). | 7-8 |
| Reporting biases | 21 | Not suitable |  |
| Certainty of evidence | 22 | Not suitable |  |
| **DISCUSSION** | | |  |
| Discussion | 23 | Hypertension is a common and frequently occurring disease, an important cause and risk factor for various cardiovascular and cerebrovascular diseases, and one of the major causes of death of cardiovascular and cerebrovascular diseases. Essential hypertension is characterized by slow onset and lack of characteristic symptoms, which are mainly higher than normal blood pressure. Its goal is to minimize the total risk of cardiovascular disease morbidity and mortality and improve physical activity and quality of life.57 In other words, it is of great significance to find other methods to control blood pressure in addition to drugs to reduce the mortality of cardiovascular diseases and improve the quality of life of residents. At present, the TCEs promoted by the General Administration of Sport of China mainly include BDJ, TJQ, YJJ and WQX, which are easy to learn, easy for patients to accept and moderate exercise intensity. Taking regulating the body, regulating breathing and nourishing the heart as the basic points, giving full play to the unique advantages of traditional Chinese medicine in dredging channels and collaterals, running qi and blood, reinforcing deficiency and strengthening body resistance, preventing and treating diseases, etc., it has achieved remarkable curative effects in the treatment of essential hypertension and gradually gained more attention and recognition.8,58  The purpose of this study was to objectively compare the effects of 4 TCEs on essential hypertension by network meta-analysis and probability ranking of 45 RCTs with SBP and DBP scores as outcomes. The results showed that TCEs significantly reduced SBP and DBP scores compared with the control group, and YJJ might be the best choice. This study provides an evidence-based medicine reference for patients with essential hypertension to choose different TCEs, and it has a certain reference value for guiding patients with essential hypertension to choose the best scheme in the future.  TJQ, BDJ, YJJ and other TCEs combine music with exercise and guide action with consciousness so that form and spirit can be interlinked, and thus spirit can reach the realm. Through manipulation exercise, the function of viscera can be stimulated and the qi, blood and body fluid can be reconciled. The internal yin and yang rise and fall orderly, and the external muscles and bones are luxuriant so that the human body tends to relax and achieve the purpose of lowering blood pressure.59 “Normalization, stability and comprehensiveness” are the three principles of treatment for patients with hypertension.7,9 Exercise therapy mainly focuses on lowering peripheral blood pressure and advocates aerobic exercise with low and medium intensity. TCEs can not only reduce blood pressure to a certain extent but also regulate the body's reaction to exercise, thus promoting the recovery of patients.57 Patients with hypertension can choose the corresponding TCEs according to their own actual situation, persistently eliminate the disease and improve their quality of life. In addition, it is also very important to prevent and treat hypertension and effectively reduce the risk of elderly individuals and chronic diseases. It is an urgent problem to popularize the concept of “prevention is more important than cure” and even to implant it into the national consciousness.  YJJ pays more attention to the exercise of posture, breathing and mind than other TCEs and exercises according to the movement of the twelve meridians and Ren and Du two Mai. That is, every potential method corresponds to dredging a meridian.60 Hong's research 41 shows that through a specific posture, the whole muscle and vein are in a highly active state, and the qi in the body is urged to move according to a specific trajectory to achieve the effect of dredging meridians, activating qi and blood, preventing diseases and keeping fit.44 Studies have also shown that exercising the vagus nerve for 1 hour in the morning can improve myocardial ischemia and hypoxia and reduce the occurrence of diseases such as hypertension and atherosclerosis, which may be related to maintaining a high level of vagus nerve tension, thus strengthening the regulation of autonomic nerve function.61 At present, there are relatively few studies on YJJ in treating essential hypertension, and its mechanism of action is not clear, which may become a breakthrough in future research.  Although this study follows the Preferred Reporting Items for Systematic Reviews and Meta-Analyses (PRISMA),62 there are still some limitations. All the included studies were published in English and Chinese, and a lack of relevant gray literature may lead to selection bias of the literature. The sample size of the included studies was small, and the quality evaluation evidence level was medium. Only 3 RCTs28,34-35 used opaque envelopes to hide the study groups. This study is difficult for blind researchers and patients, so it does not rule out the possibility of bias. The stage of hypertension, intervention period (623-48 weeks30,33,48), intervention frequency, duration of each intervention and subjectivity of outcome index measurement may affect the results of network meta-analysis. | 8-11 |
| **OTHER INFORMATION** | | |  |
| Registration and protocol | 24 |  |  |
|  |  |  |  |
|  |  |  |  |
| Support | 25 | Funding:  1.Shandong Traditional Chinese Medicine Science and Technology Development Planning (No. 2017-018);  2.Shandong University of Traditional Chinese Medicine Research and Innovation Outstanding Team (No. 220316);  3.Shandong Provincial Universities Scientific Research Development Planning (No. J18KB130). | 1 |
| Competing interests | 26 | All authors declare that they have no conflict of interests. | 11 |
| Availability of data, code and other materials | 27 | The original contributions presented in the study are included in the article/supplementary material, further inquiries can be directed to the corresponding author. |  |

*From:*  Page MJ, McKenzie JE, Bossuyt PM, Boutron I, Hoffmann TC, Mulrow CD, et al. The PRISMA 2020 statement: an updated guideline for reporting systematic reviews. BMJ 2021;372:n71. doi: 10.1136/bmj.n71

For more information, visit: <http://www.prisma-statement.org/>
